# Development of advanced humanized models: The future of pre-clinical testing of immune therapies?

**DOI:** 10.1016/j.omton.2026.201170

**Published:** 2026-03-11

**Authors:** Rebecca J. Bayliss

**Affiliations:** 1 Department of Cancer and Genetics, School of Medicine, Cardiff University, Cardiff CF14 4XN, UK; 2 Wales Applied Virology Unit, School of Medicine, Cardiff University, Cardiff CF14 4XN, UK

## Main text

Immune therapies have advanced therapeutic outcomes in a number of cancers. Future advancement of these therapies largely depends on the development of reliable immunocompetent pre-clinical models that mimic human disease.^1^ Humanized patient-derived xenograft (PDX) models have become essential for evaluating new immune therapies for clinical translation, yet their limitations restrict their use as models for advanced therapeutics.^2^ Steinkamp et al. describe a humanized mouse model addressing these limitations, supporting clinical translation of a silicified cancer cell vaccine presenting a Toll-like receptor antagonist. HLA-A-matched ovarian cancer spheroids were engrafted into a humanized mouse model to evaluate the vaccine on local immune responses and tumor progression.^3^ The outcomes of the study highlight the requirement to generate a robust and representative model for the translation of immune therapies heading to clinic. This commentary outlines the potential impact and limitations of this study.

PDX models are valuable as they retain tumor heterogeneity and genomic properties. To avoid graft rejection, PDX can be implanted orthotopically into immunodeficient mice representing a diverse range of cancer types. Although PDX is considered a more physiological model, better representative of patient responses, the use of immunodeficient mouse strains hampers the evaluation of immune therapy efficacy.^1^ Humanized mice overcome such limitations through engraftment of human-derived immune cells such as CD34^+^ hematopoietic stem cells (HSCs). Nonetheless, humanized mice present distinct challenges: MHC mismatch, high costs, and complexities in establishing models such as short experimental windows and graft-versus-host disease (GvHD).^4^ Advancements in humanized model development provide more effective, representative human systems and invaluable means to pre-clinically evaluate immunotherapies.

Steinkamp et al. develop an advanced humanized mouse model based on PDX by engrafting mice with spheroids derived from malignant ascites from a high-grade serous ovarian cancer patient. To be efficacious, silicified cancer cell vaccines require both antigen-presenting cells (APCs) and functional T cells; therefore, the authors engrafted human cord blood-derived CD34^+^ HSCs into humanized mice expressing MHC class 1 to facilitate HLA-specific T cell responses and maturation.

Initially, neonate NSG-A2 mice (MHC HLA-A2.1 allele) were irradiated before retro-orbital injection with HLA-matched HSCs. Mice commenced the study with 20% human CD45^+^ cells (12 weeks), and by 15 weeks, this had dropped from 35% to 21%. Overall, T cells increased from 5% to 19% and myeloid cells from 6% to 11%. Humanization decreased over time and was variable between mice. Despite this, mice responded to treatment in 3 of 5 vaccinated mice.

T-SNE (t-distributed stochastic neighbor embedding) analysis from ascites samples showing distinct differences between immune cell populations were identified between treated and untreated groups. Responding vaccinated mice showed increases in CD8^+^ T cells, whereas unvaccinated mice had fewer total human immune cells in the ascites. PD-1^+^ CD11b^+^ myeloid cells decreased, and fewer CD69^+^ T cells were observed in the vaccinated group, whereas PD-1 expression increased, indicative of immune exhaustion, although this was not significant between groups and did not correlate with high tumor burden.

To improve T cell responses, they developed an NSG-HLA-A2/HHD mouse model. The human leukocyte antigen (HLA)-A2.1 transgenic mouse (HHD) model expresses human MHC HLA class 1 heavy and light chains supporting maturation of T cells from HSC engraftment. Irradiated neonates were engrafted with CD34^+^ HSCs via hepatic injection. This model suffered from lower humanization levels (<20%), which likely contributed to small differences in tumor burden between groups, although fewer spheroids were detected in the ascites of vaccinated compared to unvaccinated groups. One vaccinated (41%) and one untreated (34%) mouse retained high levels of humanization. The vaccinated mouse lacked cancer spheroids compared to high levels in the control; however, the tumor signal was very low in tumor-specific tissues of vaccinated mouse, preventing definitive conclusions.

**Figure 1:**
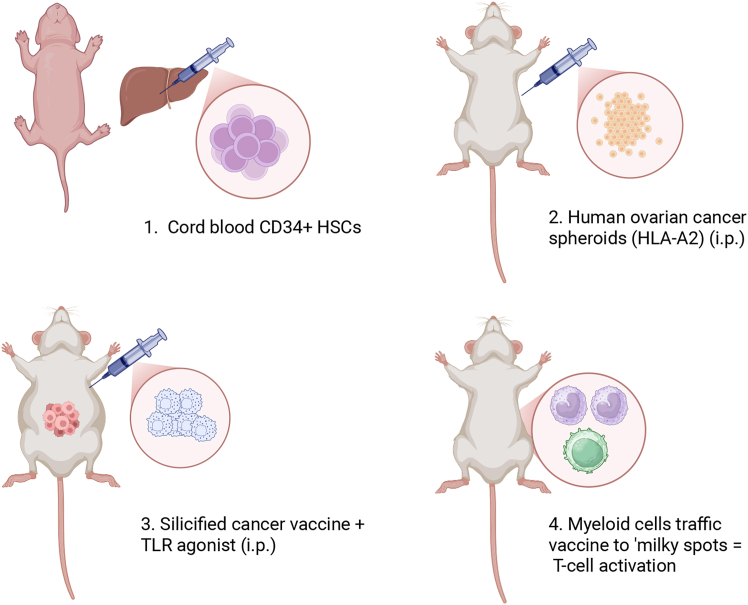
Optimal humanized mouse model NBSGW-HLA-A2/HHD (1.) NBSGW-HLA-A2/HHD neonates were humanized via intra-hepatic injection with cord blood CD34^+^ HSCs. (2.) At 15 weeks, mice were injected intraperitoneally with PDX ovarian cancer spheroids PDX-Luc2 (HLA.A2). (3.) Two doses of silicified cancer cells modified with TLR agonist were administered intraperitoneally. (4.) Tumor burden was reduced and vaccine was found to be trafficked to “milky spots” by myeloid cells resulting in localized T cell activation. Created in https://BioRender.com.

The authors therefore developed a third mouse model. To improve engraftment in NSG HLA-A2/HHD neonates, they bred HLA-A2/HHD onto the NBSGW background. NBSGW mice have a mutated *ckit* gene, enabling expression of human cytokines to support multilineage differentiation of human HSCs, omitting the need for pre-engraftment irradiation. Neonates were administered with HLA-A2.1-matched CD34^+^ HSCs, demonstrating improved engraftment compared to previous models (Figure 1). Average humanization improved to 28% (12 weeks) increasing to 37% (14 weeks). T cells increased (23%–26%) and myeloid cells decreased (8%–5%). After tumor challenge, an arrest in tumor progression was evident in vaccinated mice, while controls showed tumor progression. Another vaccinated group was treated with hematopoietic cytokine fms-like tyrosine kinase 3-ligand (FLT3-L), to support differentiation and survival of dendritic cells (DCs). FLT3-L did not enhance the CD1c^+^ DC population; nonetheless, FLT3-L-treated mice had undetectable bioluminescence in tumor-associated tissue. The authors suggest that FLT3-L caused a transient increase in APCs that boosted vaccination. To compound this, low spheroid counts were observed in the ascites of vaccinated mice compared to controls.

Ascites analysis via a 14-marker human immune panel indicted decreased CD86^+^ and CD206^+^ tumor-associated macrophages (TAMs) with increased T cells in vaccinated mice, suggestive of repolarization toward a TH1 phenotype following vaccination. Vaccine biodistribution studies using CellTrace far-red-labeled vaccine cells were associated with CD11b^+^ myeloid cells enriched at sites containing human CD45^+^ cells in adipose tissues, within immune-rich sites or “milky spots,” as demonstrated in previous *in vivo* models.

The study’s development of advanced humanized mouse models demonstrates the requirement for more representative *in vivo* models for pre-clinical translation and how sub-optimal models can hinder immune therapy development. Early humanized models are plagued by low humanization rates, instability, and MHC mismatches.^4^ The development of the NBSGW-HLA-A20/HHD model addresses such limitations, enabling improved engraftment of HSCs and stable and consistent humanization levels. Intraperitoneal engraftment of human tumor spheroids better represents the clinical scenario, as does the use of HLA-matched cells in combination with model tumors expressing human MHC HLA class 1.^3^ The development of this advanced model has broad implications for pre-clinical testing of other immune therapies undergoing IND-enabling studies.

While the study offers valuable insights, the lack of functional NK cells, B cells, and cytokines, represents a shortcoming, and with the high failure rate of immune therapies in the clinic, one could question if this model is fully representative of the human system. Do the complexity of the models and associated high costs outweigh the short duration of the experiments? Do higher humanization rates result in greater risk of GvHD? The current wider research landscape in pre-clinical testing of immune therapies cannot be ignored. With recent announcements regarding the intention of the Federal Drug Agency (FDA)^5^ and UK governments^6^ to move away from animal models for the approval of immune therapies, and with numerous research funders backing the use of alternative models, it could be argued that the development of more patient representative *ex vivo* models are required to replace humanized mouse models. Research centered around the use of patient organoids, “organs-on-chips,” and *in silico* modeling is gaining traction, as these models may ultimately replace the need for humanized mouse models.^5^

Steinkamp et al. highlight the limitations of early humanized mouse models and develop a representative human immune system to support pre-clinical evaluation of immune therapies (Figure 1). As advanced pre-clinical models evolve to create more complex and representative models of the human immune system, it increases hope that the proportion of therapies proving efficacious when translated into the clinic will increase, resulting in more effective treatment options for cancer patients.

## Acknowledgments

R.J.B. is funded by 10.13039/100012068Health and Care Research Wales awarded to Wales Applied Virology Unit.

## Author contributions

The manuscript was authored by R.J.B. The figure was illustrated by R.J.B. using Biorender.com.

## Declaration of interests

The author declares no competing interests.
